# Expression of concern: Ultrafine carbamazepine nanoparticles with enhanced water solubility and rate of dissolution

**DOI:** 10.1039/d2ra90069f

**Published:** 2022-07-21

**Authors:** Laura Fisher

**Affiliations:** Royal Society of Chemistry, Thomas Graham House, Science Park, Milton Road, Cambridge CB4 0WF UK advances-rsc@rsc.org

## Abstract

Expression of concern for ‘Ultrafine carbamazepine nanoparticles with enhanced water solubility and rate of dissolution’ by Raj Kumar *et al., RSC Adv.*, 2014, **4**, 48101–48108, https://doi.org/10.1039/C4RA08495K.

*RSC Advances* is publishing this expression of concern in order to alert our readers that we are presently unable to confirm the reliability of the data presented in the article.

The Royal Society of Chemistry was contacted by a reader who raised concerns about the reliability of the data presented in Fig. 2, 3 and S7 of the paper. The authors are unable to provide raw data to corroborate their work.

The Royal Society of Chemistry has asked the affiliated institution (Indian Institute of Technology Mandi) to investigate this matter and confirm the integrity and reliability of the data in Fig. 2, 3 and S7 of the paper. An expression of concern will continue to be associated with this manuscript until we receive information from the institution on this matter.

Laura Fisher

01/07/2022

Executive Editor, *RSC Advances*

## Supplementary Material

