# Update to expression of concern: Ultrafine carbamazepine nanoparticles with enhanced water solubility and rate of dissolution

**DOI:** 10.1039/d5ra90122g

**Published:** 2025-10-27

**Authors:** Raj Kumar, Prem Felix Siril

**Affiliations:** a School of Basic Sciences, Indian Institute of Technology Mandi Mandi-175005 Himachal Pradesh India prem@iitmandi.ac.in +91-1905-237942 +91-1905300040

## Abstract

Update to expression of concern for “Ultrafine carbamazepine nanoparticles with enhanced water solubility and rate of dissolution” by Raj Kumar *et al.*, *RSC Adv.*, 2014, **4**, 48101–48108, https://doi.org/10.1039/C4RA08495K.

The Royal Society of Chemistry published an expression of concern in order to alert our readers to the fact that concerns were raised on the reliability of the data in Fig. 2, 3 and S7. The affiliated institution (Indian Institute of Technology Mandi) was asked to investigate this matter and confirm the integrity and reliability of the data in Fig. 2, 3 and S7.

We are now able to provide the following update:

The institution investigated but was unable to confirm the integrity and reliability of the data for Fig. 2 and S7 as there is no raw data available. They recommended a correction is published for Fig. 3 and the details of this are given below. This expression of concern will continue to be associated with this article.

The authors regret mistakes in Fig. 3, where the XRD patterns corresponding to raw-griseofulvin and nano-griseofulvin were presented instead of raw-carbamazepine and nano-carbamazepine.

The XRD patterns have been recorded and plotted afresh and are shown here. The amended data are consistent with the discussions and conclusions presented.

**Fig. 1:**
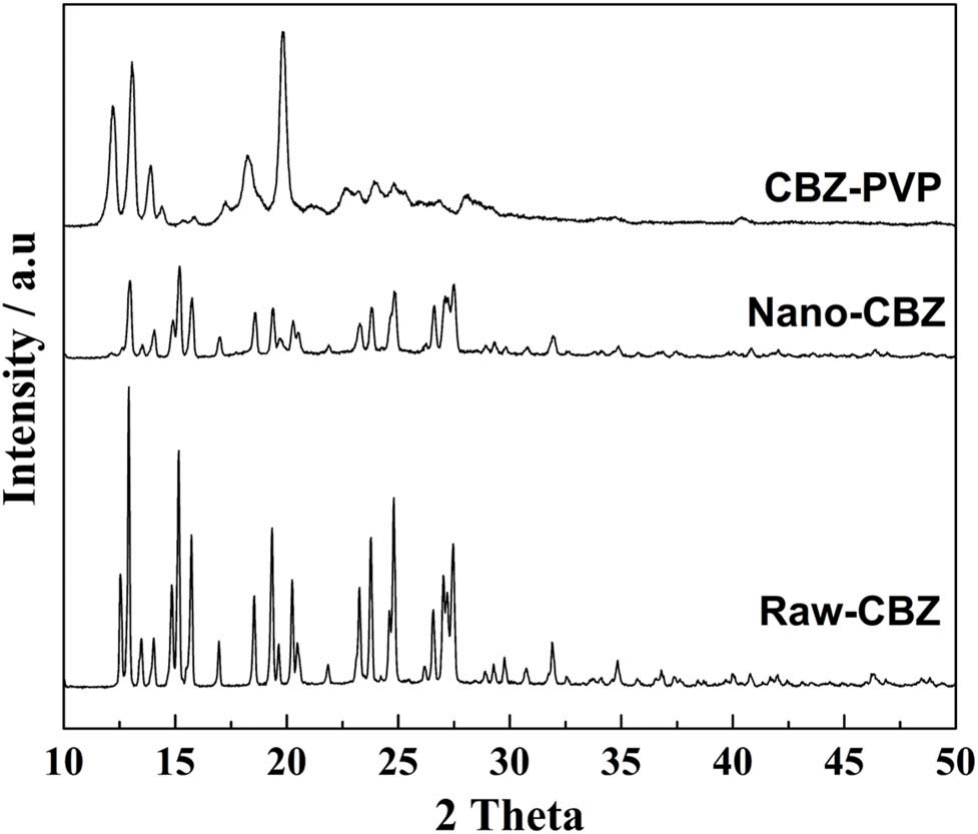
XRD patterns of raw-CBZ, nano-CBZ and CBZ–PVP.

The Royal Society of Chemistry apologises for these errors and any consequent inconvenience to authors and readers.

Laura Fisher

20th October 2025

Executive Editor, *RSC Advances*

